# Clinical Audit on ECG Monitoring in Patients Undergoing Amphetamine Withdrawal

**DOI:** 10.1192/bjo.2026.11568

**Published:** 2026-06-30

**Authors:** IQRA AIN ALI, Imtiaz Ahmad Dogar, Abdul Rehman Asgher, Fatima Tuz Zahra, Muhammad Yahya Anwaar

**Affiliations:** Faisalabad Medical University, Faisalabad, Pakistan

## Abstract

**Aims:**

Use of amphetamines is linked to serious cardiovascular events such as tachyarrhythmias, QTc prolongation, myocardial ischemia, and even sudden death from cardiac arrest. The discontinuation of these substances will increase these risks, especially because of autonomic instability and the possibility of polypharmacy with psychotropic drugs. Although the American Heart Association (AHA) recommends using the ECG when withdrawing, there is no certainty that these procedures are followed in psychiatric withdrawal units. This audit was focused on assessing the adherence to the recommended ECG monitoring guidelines in amphetamine withdrawal inpatients and determining the gaps in the current practices that require quality improvement efforts.

**Methods:**

A retrospective clinical audit was carried out at the Department of Psychiatry and Behavioral Sciences, Allied II Hospital, Faisalabad. A standardized checklist was used to examine the medical records of 70 inpatients admitted during a three-month time interval for amphetamine withdrawal, based on the AHA guidelines. The information was gathered, including demographic data, ECG performance, record of interpretation, repeat ECGs, and follow-up measures. The data were evaluated descriptively with Microsoft Excel, in which frequencies and percentages were computed to identify the adherence to each audit criterion.

**Results:**

The average age of the patients was 30.3 years (9.6), and the majority were males (97.1%). Even though all the patients confirmed taking amphetamine, only 7.1% of the patients were subjected to a baseline ECG at admission. Worryingly, none had recorded the results of ECG interpretations and QTc measurements. In addition, no repeat ECG checks or cardiac laboratory assessments were done, including the 37.1% of the patients who complained of cardiovascular symptoms. The compliance with AHA standards of monitoring was generally of low quality, which points to deep-seated flaws in the cardiac safety monitoring.

**Conclusion:**

This audit has shown that there is a distressing absence of ECG checks and recording in the treatment of amphetamine withdrawal. In order to improve patient safety, it is important to introduce mandatory baseline ECG policies and staff education on the need to monitor and incorporate ECG prompts into admission workflows. It is highly advisable that a follow-up re-audit be done between 6 and 12 months after these corrective measures are put in place in order to determine how much compliance and patient outcomes have improved. With these efforts, it will be possible to protect the cardiovascular health of patients with amphetamine withdrawal symptoms in an effective way.

